# Supplementary material for: Mass mortality of pearl oyster (Pinctada fucata (Gould)) in Japan in 2019 and 2020 is caused by an unidentified infectious agent
Source: PeerJ. 2021 Sep 21;9:e12180. doi: 10.7717/peerj.12180 (PMC8462378; doi:10.7717/peerj.12180)
Supplement: Supplemental Information 1 [file peerj-09-12180-s001.docx]

| Supplemental Table 1　Infection test by cohabitation with affected pearl oysters | | | | | | |
| --- | --- | --- | --- | --- | --- | --- |
|  | Source of infection (Donors) | |  | Mean shell scores (N=5) of recipients sampled on different days of cohabitation | | |
| Test groups | Collection site, age and number of oysters used as donors for each group | Mean shell scores (N=5) |  | 42 days | 63 days | 111 days |
| Group 1-1 | Mie Prefecture, 1+, N=4 | 4.2 ± 0.7 |  | 0.4 ± 0.4 | 1.0 ± 0.5 | 0.8 ± 0.5 |
| Group 1-2 | ditto | ditto |  | 0 ± 0 | 0.8 ± 0.5 | 0 ± 0 |
| Group 2-1 | Mie Prefecture, 2+, N=10 | 3.2 ± 0.9 |  | 0 ± 0 | 0.4 ± 0.4 | 0.4 ± 0.4 |
| Group 2-2 | ditto | ditto |  | 0 ± 0 | 0 ± 0 | 0.5 ± 0.4 |
| Negative control 1 | Ishikawa Prefecture, 2+, N=10 | 0 ± 0 |  | 0 ± 0 | 0 ± 0 | 0 ± 0 |
| Negative control 2 | ditto | ditto |  | 0 ± 0 | 0 ± 0 | 0 ± 0 |
| Healthy pearl oysters (N=20) obtained from Ishikawa Prefecture were reared with donor oysters in the same tank for each experiment. Thus, a total of 140 healthy oysters (including the healthy oysters used as donors in the control tanks) were used. | | | | | | |
|  |  |  |  |  |  |  |
