# Supplementary material for: Mass mortality of pearl oyster (Pinctada fucata (Gould)) in Japan in 2019 and 2020 is caused by an unidentified infectious agent
Source: PeerJ. 2021 Sep 21;9:e12180. doi: 10.7717/peerj.12180 (PMC8462378; doi:10.7717/peerj.12180)
Supplement: Supplemental Information 2 [file peerj-09-12180-s002.docx]

| Supplemental Table 2　Infection test by hemolymph injection | | | |
| --- | --- | --- | --- |
| Test groups | Inoculum |  | Mean shell scores of 10 surviving oysters |
| Group 1 | Supernatant of affected pearl oyster hemolymph |  | 0 ± 0 |
| Group 2 | ditto |  | 0.2 ± 0.1 |
| Negativ control 1 | Autoclaved seawater |  | 0 ± 0 |
| Negativ control 2 | ditto |  | 0 ± 0 |
| Healthy pearl oysters (N=20) obtained from Ishikawa Prefecture were used as the recipients in each group. A total of 80 healthy oysters were used. | | | |
| Supernatant of hemolymph obtained from affected pearl oysters (N=10, mean shell score: 5.0 ± 0.6) from Mie Prefecture was used as the inoculum. | | | |
